# Supplementary material for: Regulation of the macrolide resistance ABC-F translation factor MsrD
Source: Nat Commun. 2023 Jul 1;14:3891. doi: 10.1038/s41467-023-39553-8 (PMC10314930; doi:10.1038/s41467-023-39553-8)
Supplement: Supplementary file 1 — Supplementary Information [file 41467_2023_39553_MOESM1_ESM.pdf]

Supplementary information for

Regulation of the macrolide resistance ABC-F translation factor MsrD

Corentin R. Fostier[1], Farès Ousalem[1], Elodie C. Leroy[2], Saravuth Ngo[1], Heddy Soufari[2,3], C. Axel Innis[2], Yaser Hashem[2,*], Grégory Boël[1,*]

[1]Expression Génétique Microbienne, CNRS, Université Paris Cité, Institut de Biologie Physico-Chimique, 75005 Paris, France

[2]INSERM U1212 (ARNA), Institut Européen de Chimie et Biologie, Université de Bordeaux, 33607 Pessac, France

[3]Current address: NovAliX, Boulevard Sébastien Brant, Bioparc, 67405 Illkirch Cedex, France

* corresponding authors:

Grégory Boël, Institut de Biologie Physico-Chimique, 13 rue Pierre et Marie Curie, 75005 Paris, France, tel. : +33 (0) 1 58 41 51 21; e-mail: boel@ibpc.fr

Yaser Hashem, Institut Européen de Chimie et Biologie, Université de Bordeaux, 33607 Pessac, France, tel. : +33 (0) 5 40 00 88 22; e-mail: yaser.hashem@inserm.fr

Supplementary Table 1. Minimum inhibitory concentration (MIC) and half maximal inhibitory concentration (IC_{50}) of *E. coli* DB10 expressing *msrD* variants in the presence of erythromycin. See Methods for experimental details. MIC values exceeding control plasmid are shown in bold. Source data are provided as a Source Data file.

	**Erythromycin**	
***E. coli* DB10**	**MIC (µM)**	**IC_{50} (µM)**
pBAD-*Control*	2	0,179 ± 0,007
pBAD-*msrD*$_{WT}$	**16**	4,592 ± 0,582
pBAD-*msrD*$_{EQ2}$	2	0,267 ± 0,027
pBAD-*msrD*$_{E125Q}$	**4**	1,427 ± 0,147
pBAD-*msrD*$_{E434Q}$	2	0,342 ± 0,04
pBAD-*msrD*$_{\Delta Loop}$	2	0,209 ± 0,025
pBAD-*msrD*$_{\Delta PtIM}$	2	0,295 ± 0,048
pBAD-*msrD*$_{R241A}$	**16**	3,632 ± 0,316
pBAD-*msrD*$_{L242A}$	**8**	1,621 ± 0,152
pBAD-*msrD*$_{H244A}$	**8**	1,492 ± 0,126
pBAD-*msrD*$_{H244W}$	**4**	0,446 ± 0,057

Supplementary Table 2. Cryo-EM data collection and refinement statistics.

	**Erythromycin-stalled *Escherichia coli* 70S ribosome with streptococcal MsrDL nascent chain** (PDB 7Q4K, EMD-13805, EMD-13806, EMD-13807, EMD-13808)
**Data collection and processing**	
Microscope	FEI Talos Arctica (IECB, Pessac, France)
Detector	K2 Summit direct electron detector (Gatan)
Magnification (X)	120,000
Voltage (kV)	200
Electron exposure ($e^{–}/Å^2$)	64
Defocus range (μm)	-0.5 to -2.7
Pixel size (Å)	1.2
Symmetry imposed	C1
Initial particle images (no.)	158,200
Final particle images (no.)	62,093
Map resolution (Å)	70S ribosome (EMD-13805): 3<br>50S subunit (EMD-13806): 2.97<br>30S subunit Body (EMD-13807): 3.08<br>30S subunit Head (EMD-13808): 3.3
FSC threshold	0.143
**Model building and refinement**	
Initial model (PDB code)	6TC3
Model resolution (Å)	2.7
FSC threshold	0.143
Model resolution range (Å)	2.5 – 8.7
Model composition	-
Non-hydrogen atoms	146,618
Protein residues	5,682
RNA bases	4,710

Ligands	173
B-factor (Å^2)	-
Protein (min./max./mean)	31.70/183.09/69.05
Ligands (min./max./mean)	20.00/458.82/75.79
R.m.s. deviations	-
Bond lengths (Å)	0.015
Bond angles (°)	1.648
Validation	-
MolProbity score	1.49
Clashscore	3.83
Poor rotamers (%)	0.63
Ramachandran plot	-
Favored (%)	95.42
Allowed (%)	4.45
Disallowed (%)	0.13

Supplementary Table 3. Strains and plasmids used in this study.

**Bacterial strains**	**References**
E. coli DB10 (*thiA leu rna pnp gyrA rpsL* FA^{s} MLS^{s})	[1,2]
**Plasmids**	**References**
pBAD-*Control* (pBAD33)	[3]
pBAD-*msrD*$_{WT}$ (pVN50)	[3]
pBAD-*msrD*$_{EQ2}$	This study
pBAD-*msrD*$_{E125Q}$	This study
pBAD-*msrD*$_{E434Q}$	This study
pBAD-*msrD*$_{\Delta Loop}$	This study
pBAD-*msrD*$_{\Delta PtIM}$	This study
pBAD-*msrD*$_{R241A}$	This study
pBAD-*msrD*$_{L242A}$	This study
pBAD-*msrD*$_{H244A}$	This study
pBAD-*msrD*$_{H244W}$	This study
pMMB-67EH	[4]
pMMB-67EH-*yfp*	This study
pMMBpLlacO-1-67EH-*yfp*	This study
pMMB-*msrDL*-*msrD*$_{(1-3)}$*:yfp*	This study
pMMB-*msrDL*$_{(no\_ORF)}$-*msrD*$_{(1-3)}$*:yfp*	This study
pMMB-*msrDL*$_{(no\_term)}$-*msrD*$_{(1-3)}$*:yfp*	This study
pMMB-*msrDL*$_{(Y2A)}$-*msrD*$_{(1-3)}$*:yfp*	This study
pMMB-*msrDL*$_{(L3A)}$-*msrD*$_{(1-3)}$*:yfp*	This study
pMMB-*msrDL*$_{(I4A)}$-*msrD*$_{(1-3)}$*:yfp*	This study
pMMB-*msrDL*$_{(F5A)}$-*msrD*$_{(1-3)}$*:yfp*	This study
pMMB-*msrDL*$_{(M6A)}$-*msrD*$_{(1-3)}$*:yfp*	This study
pMMB-*msrDL*$_{(UAA>UGA)}$-*msrD*$_{(1-3)}$*:yfp*	This study
pMMB-*msrDL*$_{(UAA>UAG)}$-*msrD*$_{(1-3)}$*:yfp*	This study
pMMB-*msrDL*$_{(WT-isocodons)}$-*msrD*$_{(1-3)}$*:yfp*	This study
pMMB-*msrDL*$_{(MYLIFMA-isocodons)}$-*msrD*$_{(1-3)}$*:yfp*	This study

Supplementary Table 4. Oligonucleotides used in this study.

No.	Name	Sequence (5’ → 3’)	Purpose
**pBAD plasmids**			
1	msrD_F	ATGGAATTAATATTAAAAGCAAAAGACATTCGTGTGG	Amplification of *msrD*$_{WT}$
2	msrD_R	TTAGTGATGGTGATGGTGATGTTTCAGATTTATTTTCTTATC	
3	pBAD_F	CATCACCATCACCATCACTAATCTAGAGTCGACCTGCAGGC	Amplification of pBAD backbone
4	pBAD_R	GCTTTTAATATTAATTCCATGGTGAATTCCTCCTGCTAGCC	
5	msrD$_{EQ2}$_F1	GGTATTTTAGCGGATCAACCTACGAG	*msrD* mutagenesis
6	msrD$_{EQ2}$_R1	GGAAGTTACTGGGTTGATCCATTATTAG	
7	msrD$_{EQ2}$_F2	AACCCAGTAACTTCCTTGACATACC	
8	msrD$_{EQ2}$_R2	GATCCGCTAAAATACCATGAACC	
9	msrD$_{E125Q}$_F	GGTATTTTAGCGGATCAACCTACGAGCCATTTAG	
10	msrD$_{E125Q}$_R	GGCTCGTAGGTTGATCCGCTAAAATACCATG	
11	msrD$_{E434Q}$_F	CTAATAATGGATCAACCCAGTAACTTCCTTGAC	
12	msrD$_{E434Q}$_R	GGAAGTTACTGGGTTGATCCATTATTAGGATG	
13	msrD$_{\Delta Loop}$_F	GGAAAGGGCTGCGGAGGAAAAGGGAGGAGGAAAGATGTATAATGCTGCTAAAAC	
14	msrD$_{\Delta Loop}$_R	GCAGCATTATACATCTTTCCTCCTCCCTTTTCCTCCGCAGCCCTTTCCAATCGGG	
15	msrD$_{\Delta PtIM}$_F	CTGATTATCTTCGTCAGAAAGGAGGAGGACCGGAAGGCATTCGCAGAATTCG	
16	msrD$_{\Delta PtIM}$_R	CGAATTCTGCGAATGCCTTCCGGTCCTCCTCCTTTCTGACGAAGATAATCAGAATAG	
17	msrD$_{R241A}$_F	GAAGACGGAGGGGCTTTAGCTCATCAAAAATC	
18	msrD$_{R241A}$_R	GATGAGCTAAAGCCCCTCCGTCTTCAGTAC	
19	msrD$_{L242A}$_F	GACGGAGGGCGTGCAGCTCATCAAAAATCAATAG	
20	msrD$_{L242A}$_R	GATTTTTGATGAGCTGCACGCCCTCCGTCTTCAG	
21	msrD$_{H244A}$_F	GCGTTTAGCTGCTCAAAAATCAATAGGAAGTAAGG	

22	msrD$_{H244A}$_R	CTATTGATTTTTGAGCAGCTAA ACGCCCTCCGTCTTC	
23	msrD$_{H244W}$_F	GCGTTTAGCTTGGCAAAAATCA ATAGGAAGTAAGG	
24	msrD$_{H244W}$_R	CTATTGATTTTTGCCAAGCTAA ACGCCCTCCGTCTTC	
**pMMB plasmids**			
25	pMMB-PlacO_F	GTATAAATGTGAGCGGATAAC ATTGACATTGTGAGCGGATAA CAAGATACTGAGCACATCACA CAGGAAACAGAATATGTCC	Replacement of P_{tac} promoter by P_{lacO} [5]
26	pMMB-PlacO_R	GTTATCCGCTCACATTTATACA GCTCATTTCAGAATATTTGCC	
27	msrDL-msrD$_{(1-3)}$:yfp_F1	CGCAGGGTTTTCCCTGCATAC AAGCAAATGAAAGCATGCGAT TATAGACAGGAGGAAATGTTAT GGAATTAATCGTAAAAATCGTG AGCAAGG	Fusion of *msrDL-msrD*$_{(1-3)}$ cistron to *yfp*
28	msrDL-msrD$_{(1-3)}$:yfp_F2	CTGAGCACAACAATATTGGAG GAATATTTATGTATCTTATTTTC ATGTAACTCTTCCTGCTAAAAT CGCAGGGTTTTCCCTGCATAC AAGC	
29	yfp_R	TTACTTGTACAGCTCGTCCATG CCGAGAGTGATCCCGGCGGC GG	
30	pMMB-backbone_F	CATGGACGAGCTGTACAAGTA ATAATTCGAGCTCGGTACCCG GG	-
31	pMMB-backbone_R	CCTCCAATATTGTTGTGCTCAG TATCTTGTTATCCGCTCACAAT GTC	-
32	pMMB-control_F	CAACAATATTGGAGGAATATTT TAATTCGAGCTCGGTACCC	-
33	pMMB-control_R	GGGTACCGAGCTCGAATTAAA ATATTCCTCCAATATTGTTG	-
34	msrDL$_{(no_term)}$_F	ATGTATCTTATTTTCATGTAACT CTCCCTGCATACAAGCAAATG	Deletion of RIT
35	msrDL$_{(no_term)}$_R	AGAGTTACATGAAAATAAGATA CATAAATATTCCTCC	
36	msrDL$_{(no_ORF)}$_F	CAACAATATTGGAGGAATATTT TAGTATCTTATTTTCATGTAACT CTTCC	Suppression of ORF *msrDL*
37	msrDL$_{(no_ORF)}$_R	GGAAGAGTTACATGAAAATAA GATACTAAAATATTCCTCCAAT ATTGTTG	
38	msrDL$_{Y2A}$_F	CAATATTGGAGGAATATTTATG GCACTTATTTTCATGTAACTCT TCC	*msrDL* mutagenesis
39	msrDL$_{Y2A}$_R	GGAAGAGTTACATGAAAATAA GTGCCATAAATATTCCTCCAAT ATTG	
40	msrDL$_{L3A}$_F	TTGGAGGAATATTTATGTATGC AATTTTCATGTAACTCTTCC	

41	msrDL$_{L3A}$_R	GGAAGAGTTACATGAAAATTGCATACATAAATATTCCTCCAA	
42	msrDL$_{I4A}$_F	GGAGGAATATTTATGTATCTTGCATTCATGTAACTCTTCCTG	
43	msrDL$_{I4A}$_R	CAGGAAGAGTTACATGAATGCAAGATACATAAATATTCCTCC	
44	msrDL$_{F5A}$_F	GGAATATTTATGTATCTTATTGCAATGTAACTCTTCCTG	
45	msrDL$_{F5A}$_R	CAGGAAGAGTTACATTGCAATAAGATACATAAATATTCC	
46	msrDL$_{M6A}$_F	ATTTATGTATCTTATTTTCGCATAACTCTTCCTGCTAAAATCGCAGG	
47	msrDL$_{M6A}$_R	CCTGCGATTTTAGCAGGAAGAGTTATGCGAAAATAAGATACATAAAT	
48	msrDL$_{TAG}$_F	GTATCTTATTTTCATGTAGCTCTTCCTGCTAAAATCG	
49	msrDL$_{TAG}$_R	CGATTTTAGCAGGAAGAGCTACATGAAAATAAGATAC	
50	msrDL$_{TGA}$_F	GTATCTTATTTTCATGTGACTCTTCCTGCTAAAATCG	
51	msrDL$_{TGA}$_R	CGATTTTAGCAGGAAGAGTCACATGAAAATAAGATAC	
52	msrDL$_{MYLIFMA}$-isocodons_F	ATGTACCTGATCTTCATGGCCTAACTCTTCCTGCTAAAATCGCAGGG	
53	msrDL$_{MYLIFMA}$-isocodons_R	TTAGGCCATGAAGATCAGGTACATAAATATTCCTCCAATATTGTTGTGCTCAGTATCTTGTTATCCGC	
54	msrDL$_{WT}$-isocodons_F	ATTTATGTACCTGATCTTCATGTAACTCTTCCTGCTAAAATCGC	
55	msrDL$_{WT}$-isocodons_R	GCGATTTTAGCAGGAAGAGTTACATGAAGATCAGGTACATAAAT	
**In vitro transcription and translation**			
56	T7_pMMB_F	GCGAATTAATACGACTCACTATAGGGAGCGGATAACAAGATACTGAGCAC	-
57	TP_msrDL_R	GGTTATAATGAATTTTGCTTATTTAATTCCATAACATTTCCTCC	-
58	TP_NV1_R	GGTTATAATGAATTTTGCTTATT	CY5 chromophore modification in 5’
**Northern blot probe**			
59	pMMB_3UTR	CAGCCAAGCTTGCATGCCTGCAGGTCGACTCTAGAGGATCCCCGGG	-
**Cryo-EM**			
60	cryoEM-msrDL_R	GCAGGGAAAACCCTGCG	-

Supplementary Table 5. DNA templates used in this study.

No.	Name	Sequence (5' → 3')
1	TP_msrDL$_{WT}$	GCGAATTAATACGACTCACTATAGGGAGCGGATAACAAGATACTGAGCACAACAATATTGGAGGAATATTTATGTATCTTATTTTCATGTAACTCTTCCTGCTAAAATCGCAGGGTTTTCCCTGCATACAAGCAAATGAAAGCATGCGATTATAGACAGGAGGAAATGTTATGGAATTAAATAAGCAAAATTCATTATAACC
2	TP_msrDL$_{L3A}$	GCGAATTAATACGACTCACTATAGGGAGCGGATAACAAGATACTGAGCACAACAATATTGGAGGAATATTTATGTATGCAATTTTCATGTAACTCTTCCTGCTAAAATCGCAGGGTTTTCCCTGCATACAAGCAAATGAAAGCATGCGATTATAGACAGGAGGAAATGTTATGGAATTAAATAAGCAAAATTCATTATAACC
3	TP_msrDL$_{I4A}$	GCGAATTAATACGACTCACTATAGGGAGCGGATAACAAGATACTGAGCACAACAATATTGGAGGAATATTTATGTATCTTGCATTCATGTAACTCTTCCTGCTAAAATCGCAGGGTTTTCCCTGCATACAAGCAAATGAAAGCATGCGATTATAGACAGGAGGAAATGTTATGGAATTAAATAAGCAAAATTCATTATAACC
4	TP_msrDL$_{7A\text{-}iso}$	GCGAATTAATACGACTCACTATAGGGAGCGGATAACAAGATACTGAGCACAACAATATTGGAGGAATATTTATGTACCTGATCTTCATGGCCTAACTCTTCCTGCTAAAATCGCAGGGTTTTCCCTGCATACAAGCAAATGAAAGCATGCGATTATAGACAGGAGGAAATGTTATGGAATTAAATAAGCAAAATTCATTATAACC
5	CryoEM_*msrDL*	GCGAATTAATACGACTCACTATAGGGAGCGGATAACAAGATACTGAGCACAACAATATTGGAGGAATATTTATGTATCTTATTTTCATGTAACTCTTCCTGCTAAAATCGCAGGGTTTTCCCTGC

SUPPLEMENTARY FIGURE 1

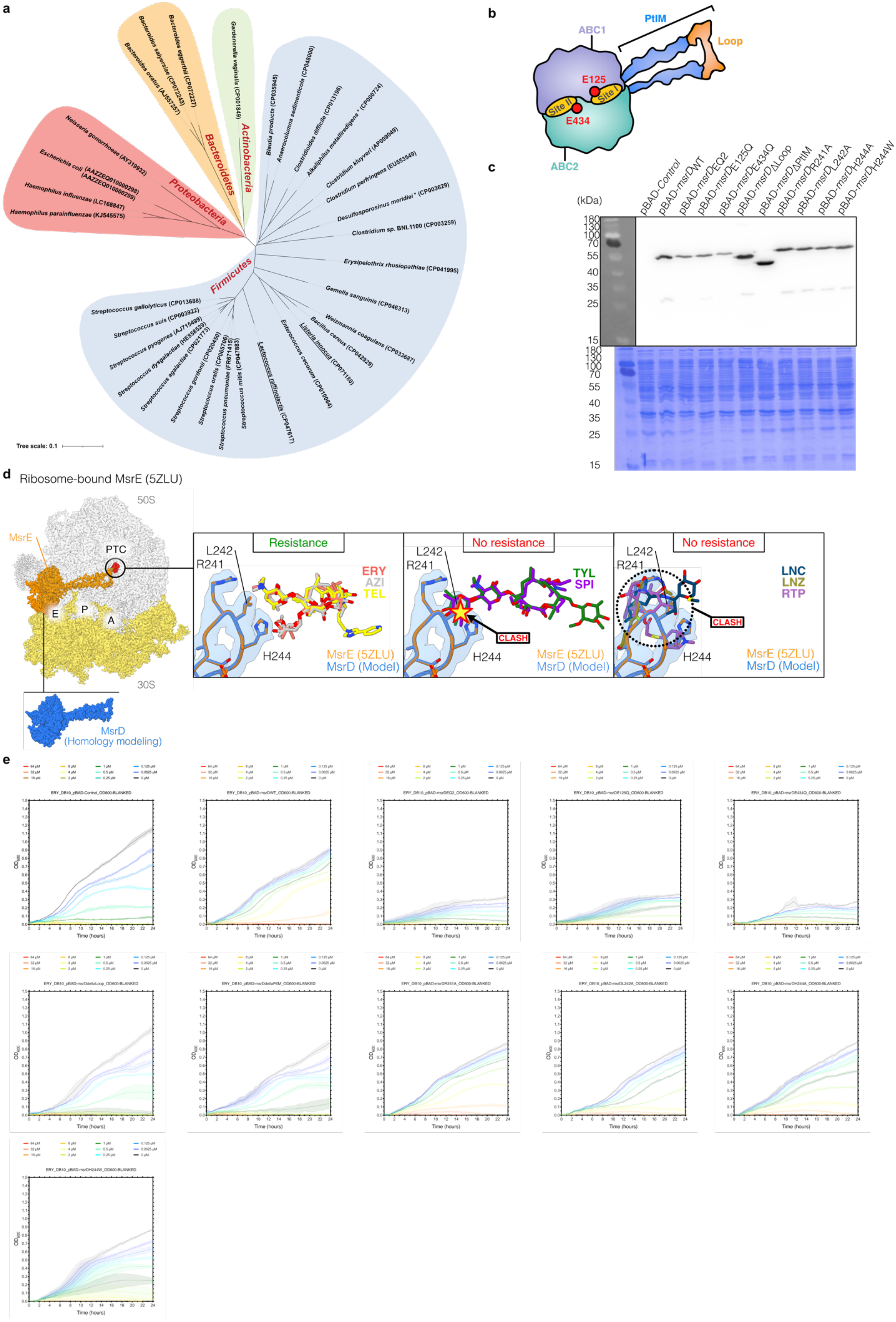

Supplementary figure 1. *In vivo* characterization of MsrD. (**a**) Dissemination of the *mefA/msrD* macrolide resistance operon among a wide range of non-pathogenic and pathogenic bacterial species. The *mefA/msrD* operon (Genbank accession No. FR671415) was blasted and identified in indicated species, integrated to the genome. Species where the operon was found on plasmid are underlined. Species where the ORF *msrDL* and the gene *msrD* were found to disseminate in absence of the first part of the operon (containing *mefAL* and *mefA*) are indicated by an asterisk. Corresponding Genebank accession numbers are indicated between brackets. To generate the tree, 16S rRNA sequences were retrieved, aligned using Clustal W[6], and resulting cladogram was adapted on iTOL (https://itol.embl.de/). Colored zones indicate bacterial phyla. (**b**) Schematic of MsrD illustrating main features of ABC-F translation factors and the position of ATP hydrolysis Site I and Site II whose catalytic residues are respectively E125 and E434. Colors are the same as Fig. 1b. (**c**) Expression level of the different MsrD variants assessed by western blot. Bacteria were grown in MH medium in the presence of 0.2 % L-Arabinose for 24 h at 37 °C under agitation before harvesting. Cells were resuspended in Laemmli buffer and normalized relative to OD_{600}. On the top the western blot is presented and on the bottom, the Coomassie stained membrane as loading control. (**d**) Steric occlusion mechanism by MsrD is incompatible with its resistance profile. A homology model of MsrD was generated using SWISS-MODEL [7] and aligned to ribosome-bound MsrE structure (PDB 5ZLU) [8]. Antibiotics to which MsrD provides resistance (ERY PDB: 6ND6, red; AZI PDB: 4V7Y, gray; TEL PDB: 4V7Z, yellow) or not (TYL PDB: 1K9M, green; SPI PDB: 1KD1, violet; LNC PDB: 5HKV, dark teal; LNZ PDB: 3CPW, khaki; RTP PDB: 2OGO, purple) were aligned based on domain V of 23S rRNA [9–14]. Density for MsrE is shown in pale blue, and conserved residues R241A, L242A and H244A are indicated. (**e**) Growth curves of *E. coli* DB10 strains expressing *msrD* variants in the presence of different concentration of ERY. The curves represent the mean of 3 independent measurements with the SD. These curves were used to generate Fig. 1c. Source data are provided as a Source Data file**.**

SUPPLEMENTARY FIGURE 2

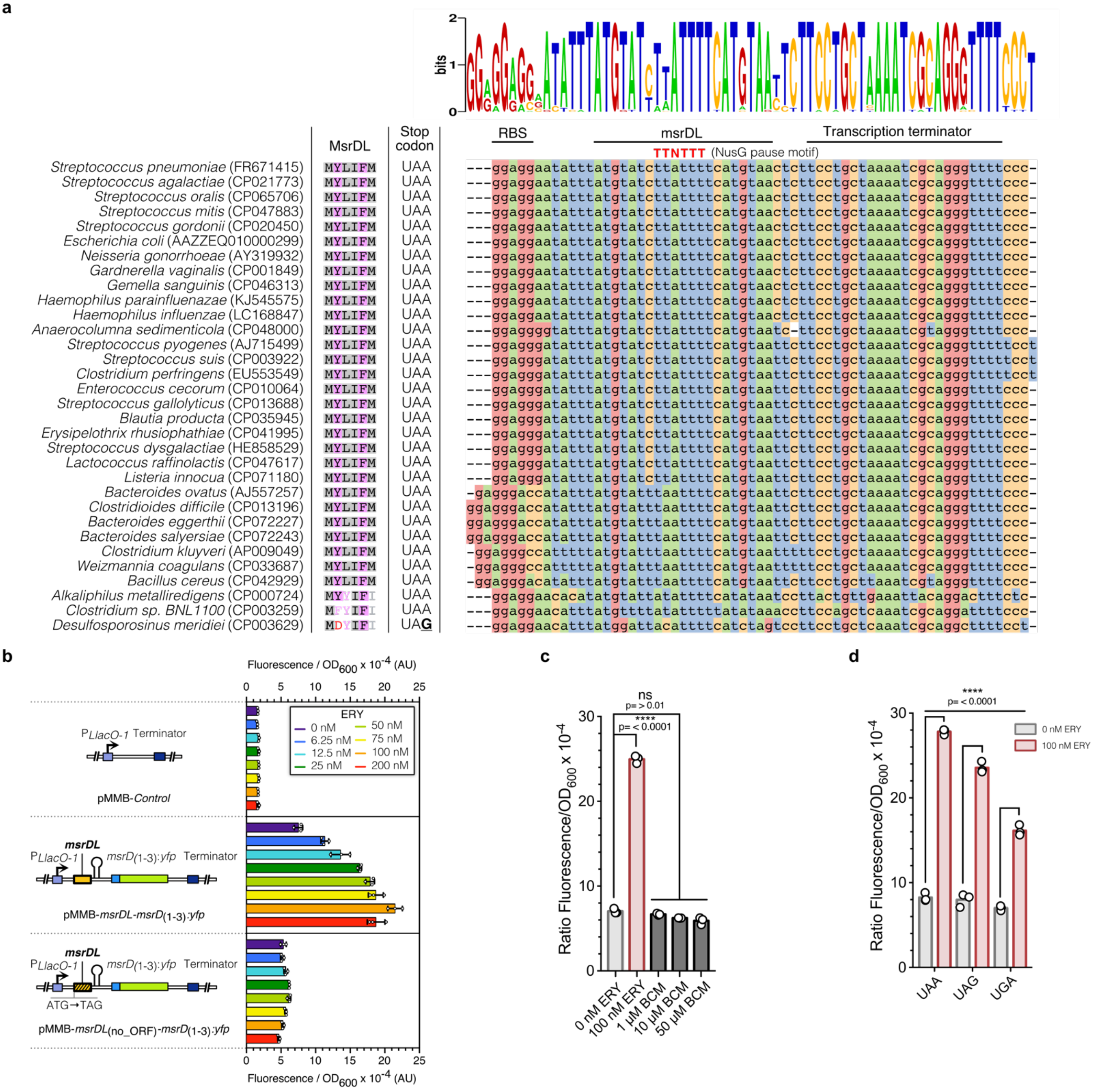

Supplementary figure 2. Rho-independent transcription termination regulates *msrD* expression. (**a**) Conservation of *msrDL*, NusG-dependent RNAP pausing site and its rho-independent transcription terminator. Sequences corresponding to Fig. 1a were aligned with Clustal W [6], and visualized with JalView according to nucleotide [15]. Genbank accession numbers are indicated between brackets. Logo was generated using WebLogo (https://weblogo.berkeley.edu/logo.cgi). (**b**) Raw OD_{600} and fluorescence measurements presented on Fig. 1b. (**c**) Bicyclomycin (BCM) failed to constitutively induce *msrD*$_{(1-3)}$:*yfp* expression in absence of ERY after 17 h, demonstrating that *msrD* is not regulated by a Rho-dependent terminator. The *p* values for the comparison with the 0 mM ERY condition where: <0.0001 for 100 nM ERY, 0.127 for 1 µM BCM, 0.01 for 10 µM BCM and 0.02 for 1 µM BCM. (**d**) Effects of *msrDL* stop codon mutation on the expression of *msrD*$_{(1:3)}$:*yfp*. Bacteria were grown during 17 h in presence of 1 mM IPTG, in the absence (grey histograms) or in the presence of 100 nM ERY (red histograms). The *p* value for the comparison between the 0 mM and 100 nM ERY conditions for all the tested stop codons were under 0.0001. (**c** and **d**) Error bars represent mean ± s.d. for triplicate experiments and the *p* values were determined by unpaired two-sided *t*-test without adjustments. Source data are provided as a Source Data file**.**

SUPPLEMENTARY FIGURE 3

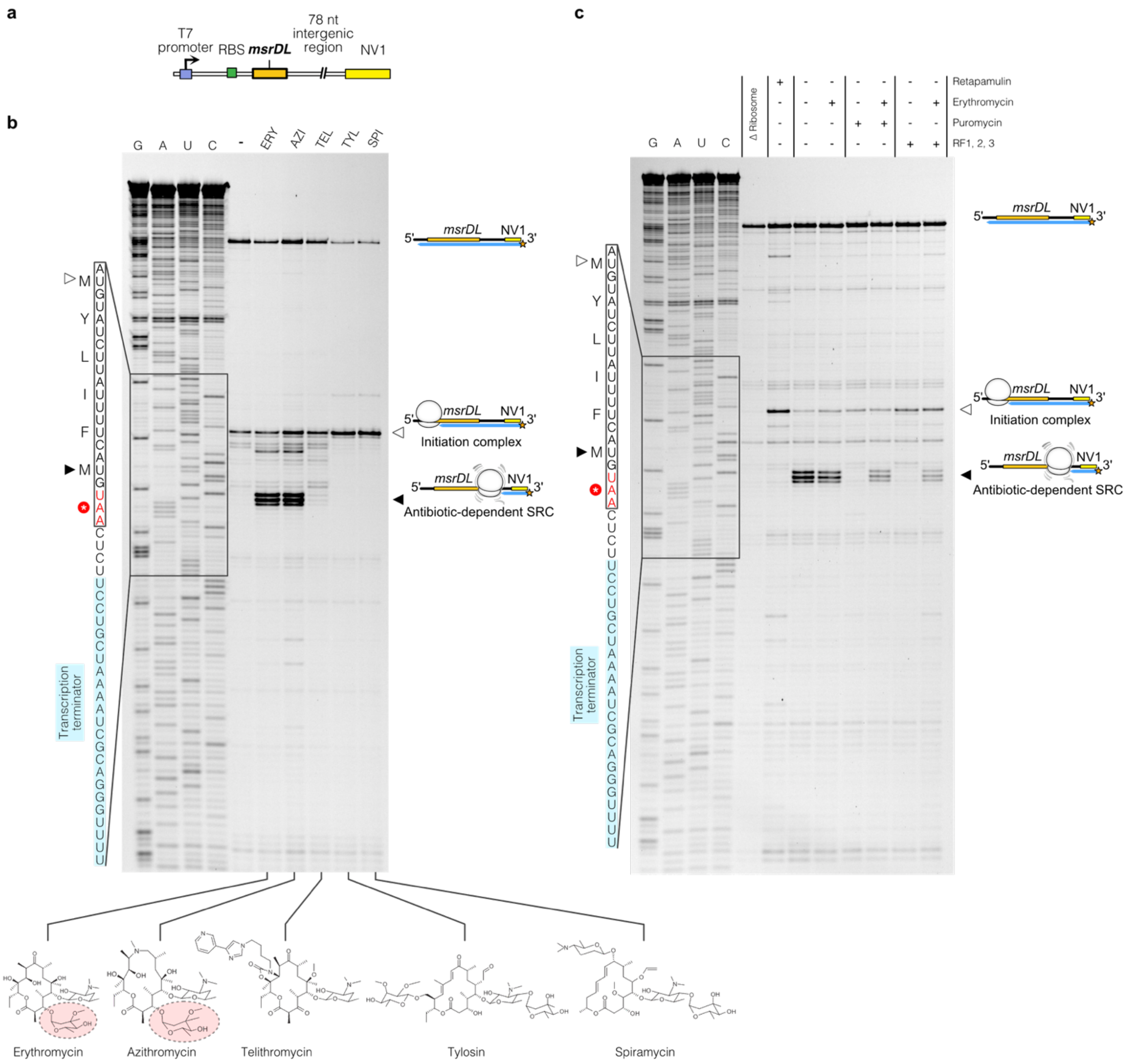

Supplementary figure 3. Biochemical characterization of MsrDL mode of action. (**a**) Schematic of the matrix TP_msrDL$_{WT}$ (Supplementary Table 5) used to generate synthetic mRNAs for *in vitro* experiments (RBS, ribosome binding site; NV1, annealing site for CY5-labelled primer). (**b and c**) Uncropped toe-printing gels presented in Fig. 3a and 3c. Formation of MsrDL-SRC would occlude formation of the Rho-independent transcription terminator (shown in light blue).

SUPPLEMENTARY FIGURE 4

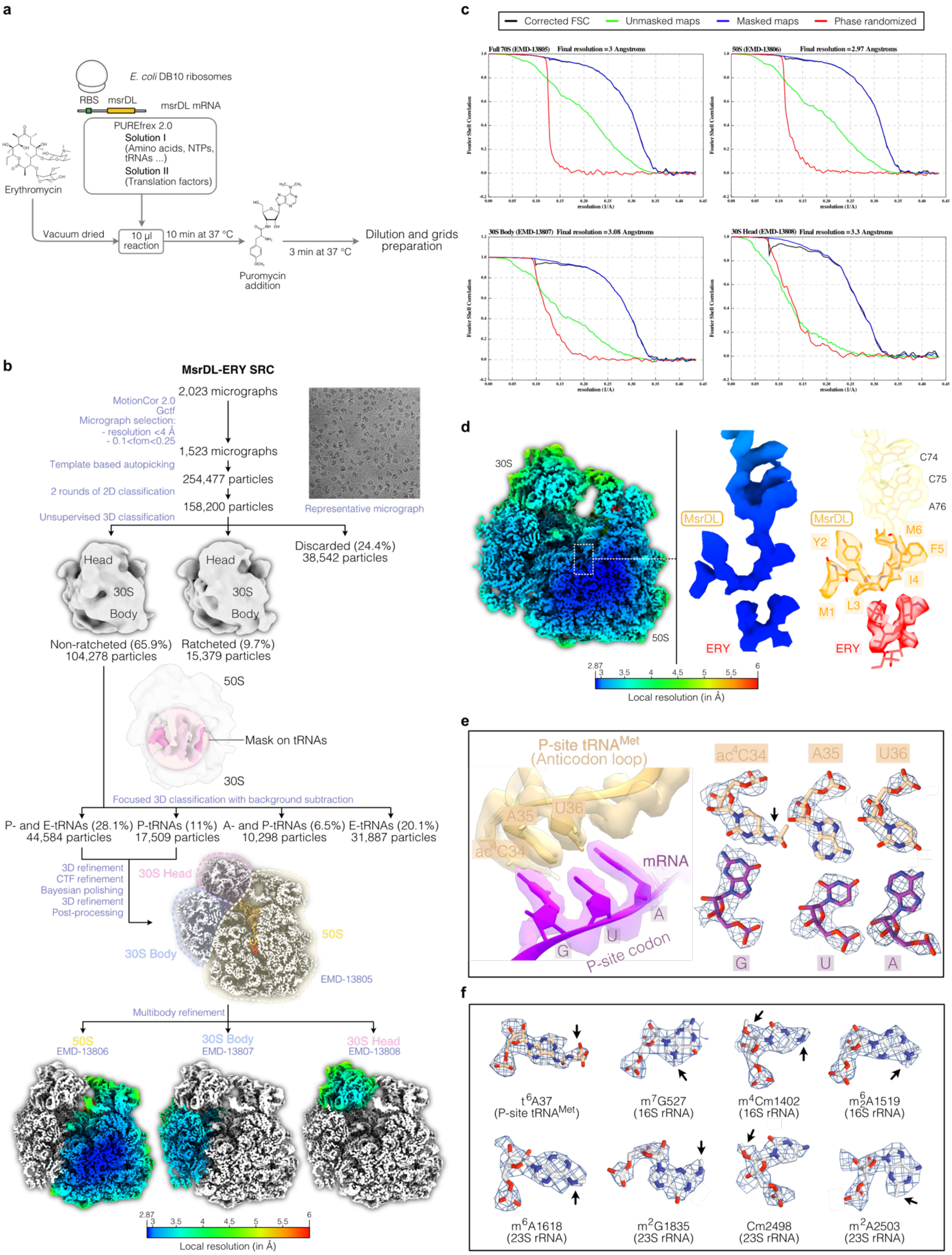

Supplementary figure 4. Generation of the cryo-EM sample, data processing and model building. (**a**) Workflow for the generation of cryo-EM sample. See Methods for details. (**b**) Cryo-EM data processing workflow as described in the Methods. EMD accession numbers are indicated for each map. Maps obtained after multibody refinement are shown as transverse sections and colored according to local resolution. (**c**) Fourier Shell Correlation (FSC) curves of the 70S ribosome and the maps obtained after multibody refinement. (**d**) Transverse section of the composite map obtained after multibody refinement and isolated densities for MsrDL-tRNA and ERY, colored according to local resolution. The corresponding atomic reconstruction of the nascent chain is shown beside. (**e**) Details of the mRNA codon-tRNA anticodon interactions and corresponding densities for each nucleotide. (**f**) Details of clearly identifiable post-transcriptional modifications and corresponding densities.

SUPPLEMENTARY FIGURE 5

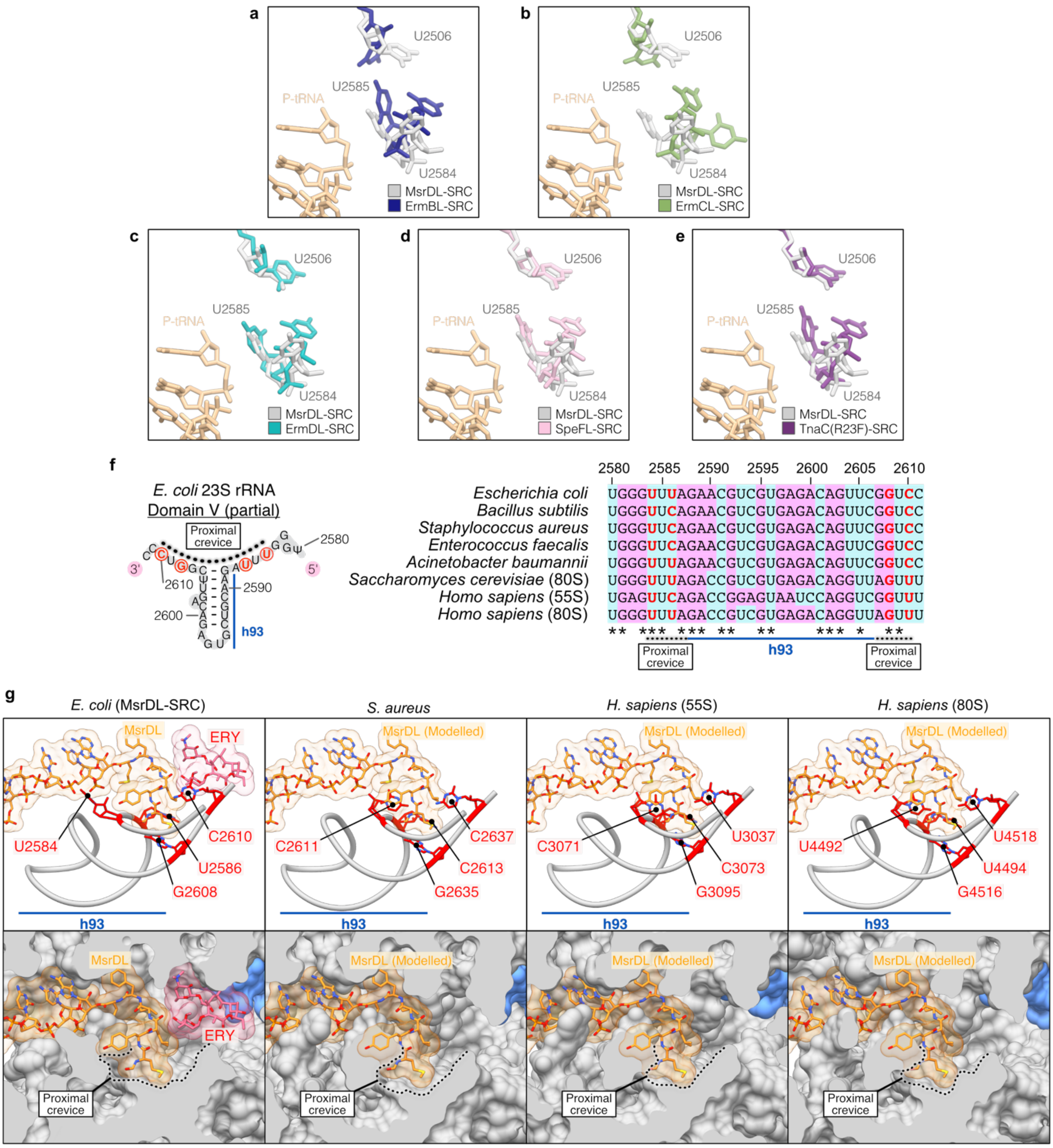

Supplementary figure 5. MsrDL engages within a universally conserved crevice at the NPET entrance. (**a to e**) Comparison of the conformation of 23S rRNA bases U2506, U2584 and U2585 for MsrDL-, ErmBL-, ErmCL-, ErmDL-, SpeFL- and TnaC(R23F)-stalled ribosomes structures [15–19](respectively PDB: 5JTE, 3J7Z, 7NSO, 6TC3, 7O1A). Structures were aligned based on domain V of 23S rRNA. (**f**) Sequence conservation of the proximal crevice between far-related species. The diagram shows a part of 23S rRNA domain V of *E. coli* and the location of proximal crevice at the base of h93. Large subunit rRNAs were aligned using Clustal W[5] and visualized with Jalview[14] (purines, purple; pyrimidines, teal). Bases delimitating the proximal crevice (U2584, U2586, G2608 and C2610) are highlighted in red. Nucleotides numbering is relative to *E. coli* 23S rRNA sequence. (**g**) Structural conservation of the proximal crevice between far-related species. Top, cartoon representation of h93 and proximal crevice in *E. coli*, *S. aureus*, *H. sapiens* 55S mitoribosome and 80S cytosolic ribosome [20–22] (respectively PDB: 6YEF, 7A5F, 6OLI). Bases delimitating the proximal crevice are highlighted in red and numbered relative to the considered specie. Bottom, sagittal cut of ribosome tunnel shown on top panels depicted as surface. Ribosomal protein uL4 is shown in pale blue. Structures were aligned based on domain V of 23S rRNA.

SUPPLEMENTARY FIGURE 6

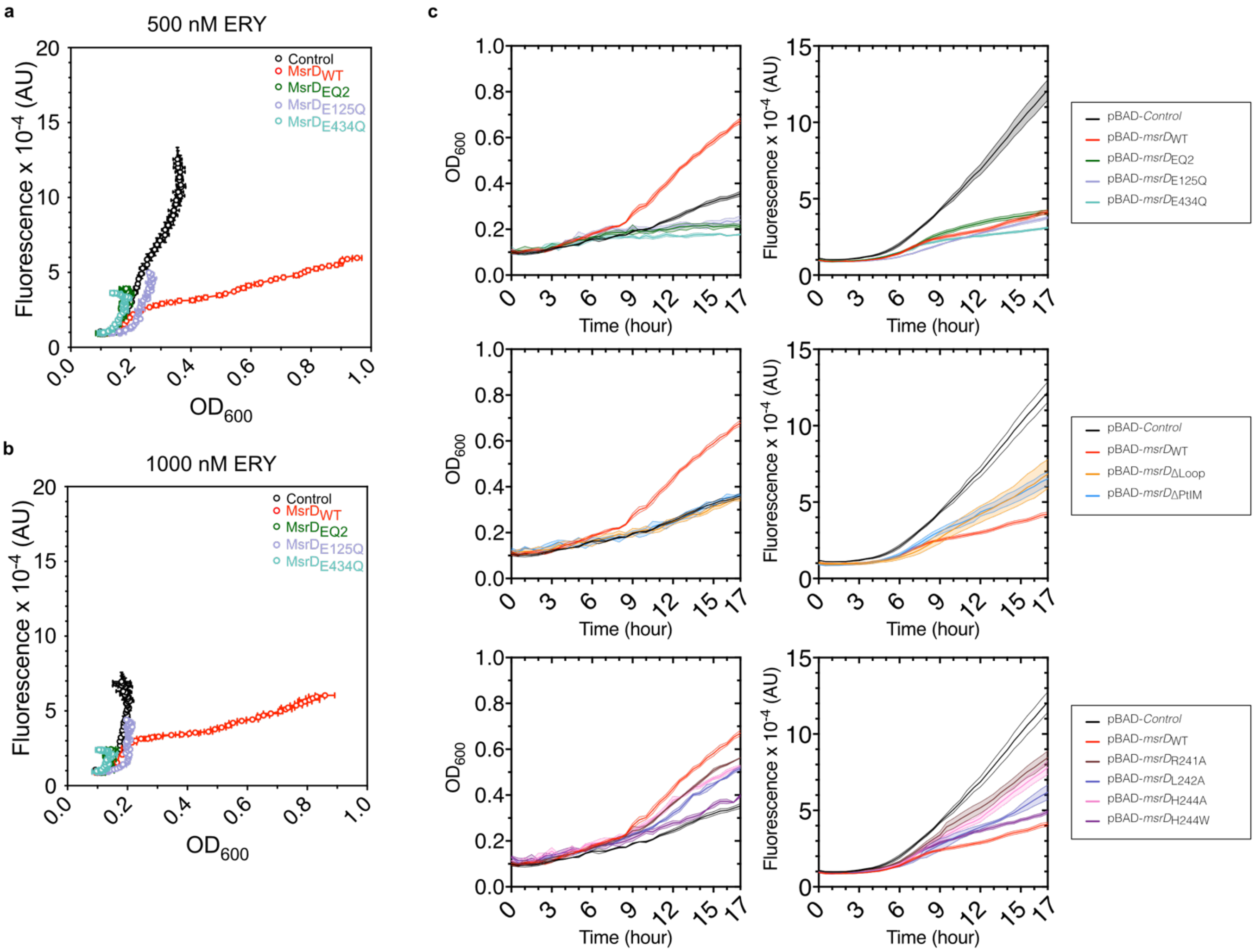

Supplementary figure 6. MsrD negatively regulates its own synthesis upon erythromycin exposure. Effects of MsrD variants on MsrDL. *E. coli* DB10 containing pMMB-*msrDL-msrD*(1-3)*:yfp* and expressing various *msrD* mutants were grown in presence of 0.2 % L-Arabinose, 1 mM IPTG and different ERY concentration. (**a and b**) Experiments done in presence of 500 nM (**a**) and 1000 nM (**b**) of ERY, fluorescence was plotted against OD_{600}, error bars for both axes represent mean ± s.d. for triplicate experiments. (**c**) Raw data of the experiment done at 300 nM and presented in Fig. 6 both OD_{600} (right) and fluorescence (left) being recorded over 17 h. (**a-c**) Error bars for both axes represent mean ± s.d. for triplicate experiments. Source data are provided as a Source Data file**.**

SUPPLEMENTARY REFERENCES

1. Datta, N., Hedges, R. W., Becker, D. & Davies, J. Plasmid-determined Fusidic Acid Resistance in the Enterobacteriaceae. *Microbiology,* **83**, 191–196 (1974).
2. Arthur, M. & Courvalin, P. Contribution of two different mechanisms to erythromycin resistance in Escherichia coli. *Antimicrob Agents Chemother* **30**, 694–700 (1986).
3. Nunez-Samudio, V. & Chesneau, O. Functional interplay between the ATP binding cassette Msr(D) protein and the membrane facilitator superfamily Mef(E) transporter for macrolide resistance in Escherichia coli. *Research in Microbiology* **164**, 226–235 (2013).
4. Fürste, J. P. *et al.* Molecular cloning of the plasmid RP4 primase region in a multi-host-range tacP expression vector. *Gene* **48**, 119–131 (1986).
5. Lutz, R. & Bujard, H. Independent and Tight Regulation of Transcriptional Units in Escherichia Coli Via the LacR/O, the TetR/O and AraC/I1-I2 Regulatory Elements. *Nucleic Acids Res* **25**, 1203–1210 (1997).
6. Larkin, M. A. *et al.* Clustal W and Clustal X version 2.0. *Bioinformatics* **23**, 2947–2948 (2007).
7. Waterhouse, A. *et al.* SWISS-MODEL: homology modelling of protein structures and complexes. *Nucleic Acids Res* **46**, W296–W303 (2018).
8. Su, W. *et al.* Ribosome protection by antibiotic resistance ATP-binding cassette protein. *PNAS* **115**, 5157–5162 (2018).
9. Bulkley, D., Innis, C. A., Blaha, G. & Steitz, T. A. Revisiting the structures of several antibiotics bound to the bacterial ribosome. *PNAS* **107**, 17158–17163 (2010).
10. Davidovich, C. *et al.* Induced-fit tightens pleuromutilins binding to ribosomes and remote interactions enable their selectivity. *PNAS* **104**, 4291–4296 (2007).
11. Hansen, J. L. *et al.* The Structures of Four Macrolide Antibiotics Bound to the Large Ribosomal Subunit. *Molecular Cell* **10**, 117–128 (2002).
12. Ippolito, J. A. *et al.* Crystal Structure of the Oxazolidinone Antibiotic Linezolid Bound to the 50S Ribosomal Subunit. *J. Med. Chem.* **51**, 3353–3356 (2008).
13. Matzov, D. *et al.* Structural insights of lincosamides targeting the ribosome of Staphylococcus aureus. *Nucleic Acids Research* **45**, 10284–10292 (2017).
14. Svetlov, M. S. *et al.* High-resolution crystal structures of ribosome-bound chloramphenicol and erythromycin provide the ultimate basis for their competition. *RNA* **25**, 600–606 (2019).
15. Waterhouse, A. M., Procter, J. B., Martin, D. M. A., Clamp, M. & Barton, G. J. Jalview Version 2—a multiple sequence alignment editor and analysis workbench. *Bioinformatics* **25**, 1189–1191 (2009).
16. Arenz, S. *et al.* Drug Sensing by the Ribosome Induces Translational Arrest via Active Site Perturbation. *Molecular Cell* **56**, 446–452 (2014).
17. Arenz, S. *et al.* A combined cryo-EM and molecular dynamics approach reveals the mechanism of ErmBL-mediated translation arrest. *Nat Commun* **7**, (2016).
18. Beckert, B. *et al.* Structural and mechanistic basis for translation inhibition by macrolide and ketolide antibiotics. *Nat Commun* **12**, 4466 (2021).
19. Herrero del Valle, A. *et al.* Ornithine capture by a translating ribosome controls bacterial polyamine synthesis. *Nature Microbiology* **5**, 554–561 (2020).
20. van der Stel, A.-X. *et al.* Structural basis for the tryptophan sensitivity of TnaC-mediated ribosome stalling. *Nat Commun* **12**, 5340 (2021).
21. Desai, N. *et al.* Elongational stalling activates mitoribosome-associated quality control. *Science* **370**, 1105–1110 (2020).
22. Golubev, A. *et al.* Cryo-EM structure of the ribosome functional complex of the human pathogen Staphylococcus aureus at 3.2 Å resolution. *FEBS Letters* **594**, 3551–3567 (2020).
23. Li, W. *et al.* Structural basis for selective stalling of human ribosome nascent chain complexes by a drug-like molecule. *Nat Struct Mol Biol* **26**, 501–509 (2019).
