# Supplementary material for: Regulation of the macrolide resistance ABC-F translation factor MsrD
Source: Nat Commun. 2023 Jul 1;14:3891. doi: 10.1038/s41467-023-39553-8 (PMC10314930; doi:10.1038/s41467-023-39553-8)
Supplement: Supplementary file 3 — Reporting Summary [file 41467_2023_39553_MOESM3_ESM.pdf]

nature portfolio

Corresponding author(s): Gregory Boel
Last updated by author(s): Jun 13, 2023

Reporting Summary

Nature Portfolio wishes to improve the reproducibility of the work that we publish. This form provides structure for consistency and transparency in reporting. For further information on Nature Portfolio policies, see our Editorial Policies and the Editorial Policy Checklist.

Statistics

For all statistical analyses, confirm that the following items are present in the figure legend, table legend, main text, or Methods section.

n/a	Confirmed	
☐	☒	The exact sample size (*n*) for each experimental group/condition, given as a discrete number and unit of measurement
☐	☒	A statement on whether measurements were taken from distinct samples or whether the same sample was measured repeatedly
☐	☒	The statistical test(s) used AND whether they are one- or two-sided<br>*Only common tests should be described solely by name; describe more complex techniques in the Methods section.*
☒	☐	A description of all covariates tested
☒	☐	A description of any assumptions or corrections, such as tests of normality and adjustment for multiple comparisons
☐	☒	A full description of the statistical parameters including central tendency (e.g. means) or other basic estimates (e.g. regression coefficient) AND variation (e.g. standard deviation) or associated estimates of uncertainty (e.g. confidence intervals)
☐	☒	For null hypothesis testing, the test statistic (e.g. *F*, *t*, *r*) with confidence intervals, effect sizes, degrees of freedom and *P* value noted<br>*Give P values as exact values whenever suitable.*
☒	☐	For Bayesian analysis, information on the choice of priors and Markov chain Monte Carlo settings
☒	☐	For hierarchical and complex designs, identification of the appropriate level for tests and full reporting of outcomes
☒	☐	Estimates of effect sizes (e.g. Cohen's *d*, Pearson's *r*), indicating how they were calculated

Our web collection on statistics for biologists contains articles on many of the points above.

Software and code

Policy information about availability of computer code

Data collection

For the structural part of the manuscriptwe used SerialEM software (3.8 version) for
automated data acquisition of the micrographs

Data analysis

MotionCor2 (Linux1 .4.5) was used for dose weighting, drift and whole-frame motion correction. A dose weighted average image of the stack was used to
determine the contrast transfer function with the software Gctf. Classification was built in RELION 3.
The local resolution was estimated using ResMap (Linux 64 bits, version 1.1.4). Figures depicting molecular structures or electronic density maps
were prepared using PyMOL Molecular Graphies System (v1.8.4), Chimera UCSF (V1.2) and ChimeraX (V1.4) 93,94. The atomic model was refined
using phenix 1.16-3649 and coot 0.8.9.2.
Growth curves, histograms and polyribosomes profiles were generated using GraphPad Prism 7 {GraphPad). Sequence alignments were visualized
with JalView. Western blotting, northern blotting and toe-printing gels were analyzed using Fiji (2.0.0 rc-59/151k).
Ali the details and reference are included in the methods section of our manuscript.

For manuscripts utilizing custom algorithms or software that are central to the research but not yet described in published literature, software must be made available to editors and reviewers. We strongly encourage code deposition in a community repository (e.g. GitHub). See the Nature Portfolio guidelines for submitting code & software for further information.

Data

Policy information about availability of data

All manuscripts must include a data availability statement. This statement should provide the following information, where applicable:

- Accession codes, unique identifiers, or web links for publicly available datasets
- A description of any restrictions on data availability
- For clinical datasets or third party data, please ensure that the statement adheres to our policy

Cryo-EM map of erythromycin-stalled Escherichia coli 70S ribosome with streptococcal MsrDL nascent chain has been deposited at the Electron Microscopy Data Bank (EMDB) with accession code EMD-13805, as well as 50S, 30S Body and 30S Head maps obtained after multibody refinement with accession code EMD-13806, EMD-13807, EMD-13808 respectively. Corresponding atomic model has been deposited in the Protein Data Bank (PDB) with accession code 7O4K. The msrD sequence is accessible in Genebank database under the No. FR671415. All the other sequences use for the sequence alignment in Supplementary Figures 1 and 2 were extracted from Genebank database, the references No. are indicated in the Supplementary Figure 2.

Field-specific reporting

Please select the one below that is the best fit for your research. If you are not sure, read the appropriate sections before making your selection.

☒ Life sciences ☐ Behavioural & social sciences ☐ Ecological, evolutionary & environmental sciences

For a reference copy of the document with all sections, see nature.com/documents/nr-reporting-summary-flat.pdf

Life sciences study design

All studies must disclose on these points even when the disclosure is negative.

Sample size	All the presented experiments were carry out in triplicate as a standard in the field. Statistical analyses showed significant differences between tested conditions, confirming that the sample sizes were sufficient
Data exclusions	No data exclusion
Replication	All experiments were replicated. All attempts at replication were successful.
Randomization	Randomization was not relevant for this study, since the outcomes of the experiments are not dependent of the judgment of the researcher but depend of control reactions.
Blinding	Blinding was not relevant for this study, since the outcomes of the experiments are not dependent of the judgment of the researcher but depend of control reactions.

Reporting for specific materials, systems and methods

We require information from authors about some types of materials, experimental systems and methods used in many studies. Here, indicate whether each material, system or method listed is relevant to your study. If you are not sure if a list item applies to your research, read the appropriate section before selecting a response.

Materials & experimental systems

n/a	Involved in the study	
☐	☒	Antibodies
☒	☐	Eukaryotic cell lines
☒	☐	Palaeontology and archaeology
☒	☐	Animals and other organisms
☒	☐	Human research participants
☒	☐	Clinical data
☒	☐	Dual use research of concern

Methods

n/a	Involved in the study	
☒	☐	ChIP-seq
☒	☐	Flow cytometry
☒	☐	MRI-based neuroimaging

Antibodies

Antibodies used	C-term His-tagged MsrD variants were detected using anti-6xHis-tag primary antibody (Covalab HIS.HS/ EH158) at a 1:2 000 dilution in lX PBS, 0.1 % Tween-20) combined with anti-mouse-HRP secondary antibody {Covalab, lab0252) at 1:20 000 dilution in lX PBS, 0.1 % Tween-20).
Validation	We carried out Western blot on a protein lysates from strains that express or not MsrD-6His (Supplementary Fig. le). A specific band

Validation

at the right size was detected only in the strain expressing MsrD-6His.
