# Supplementary material for: Regulation of the macrolide resistance ABC-F translation factor MsrD
Source: Nat Commun. 2023 Jul 1;14:3891. doi: 10.1038/s41467-023-39553-8 (PMC10314930; doi:10.1038/s41467-023-39553-8)

Uncropped gels presented in the manuscript: Regulation of the macrolide resistance ABC-F translation factor MsrD by Fostier *et al*.,

Figure 3a uncropped gel

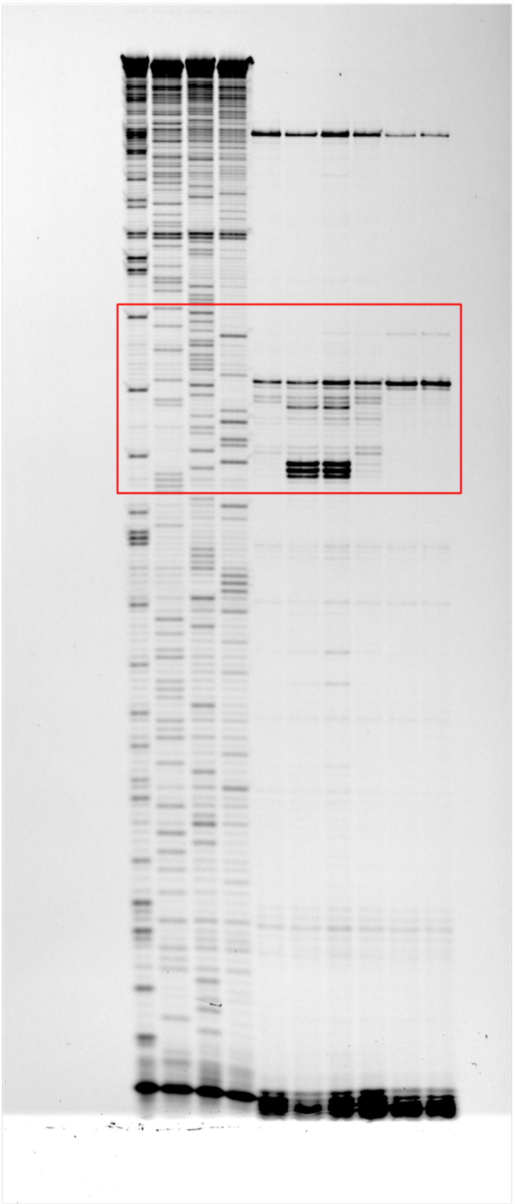

Figure 3c uncropped gel

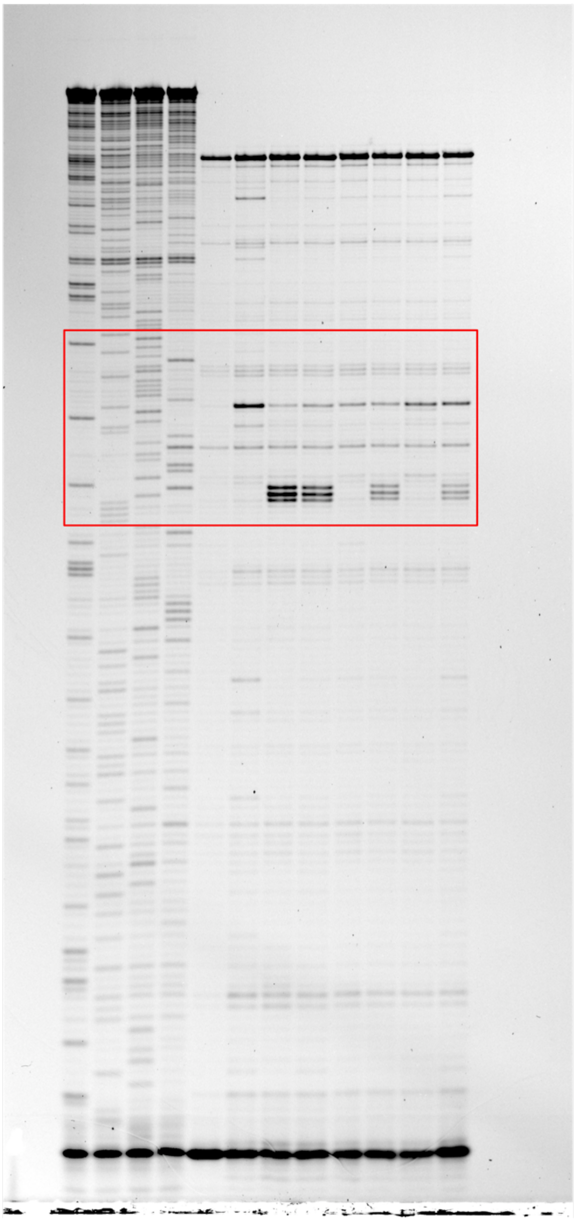

Figure 3f uncropped gel

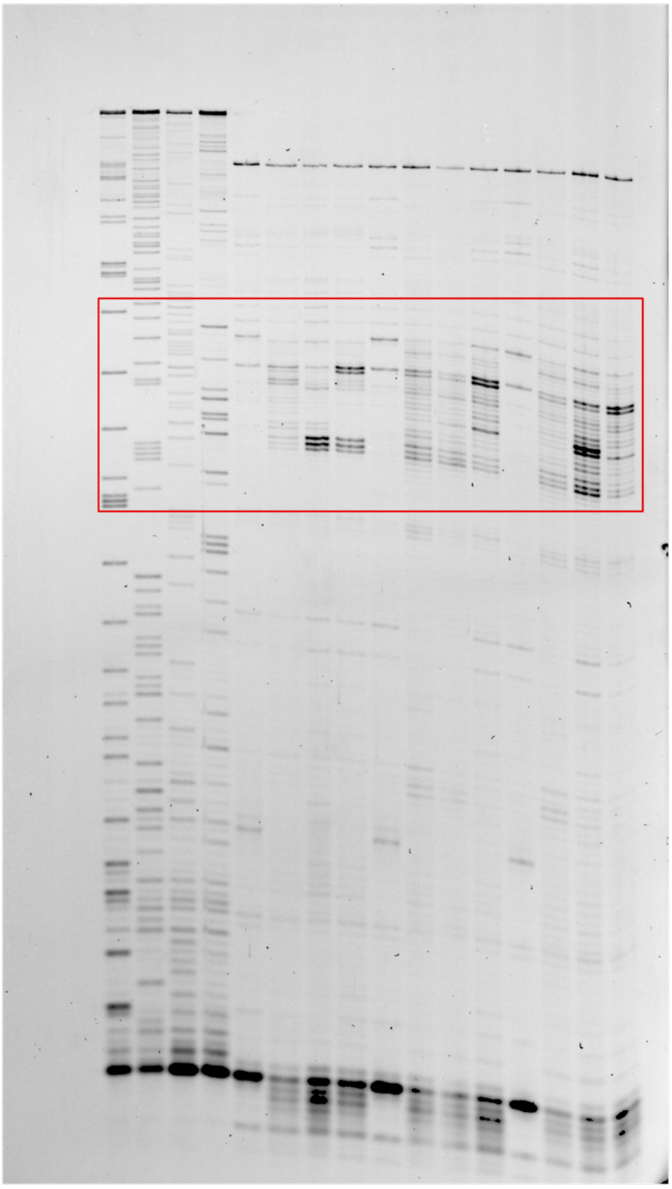

Figure 3e uncropped gel

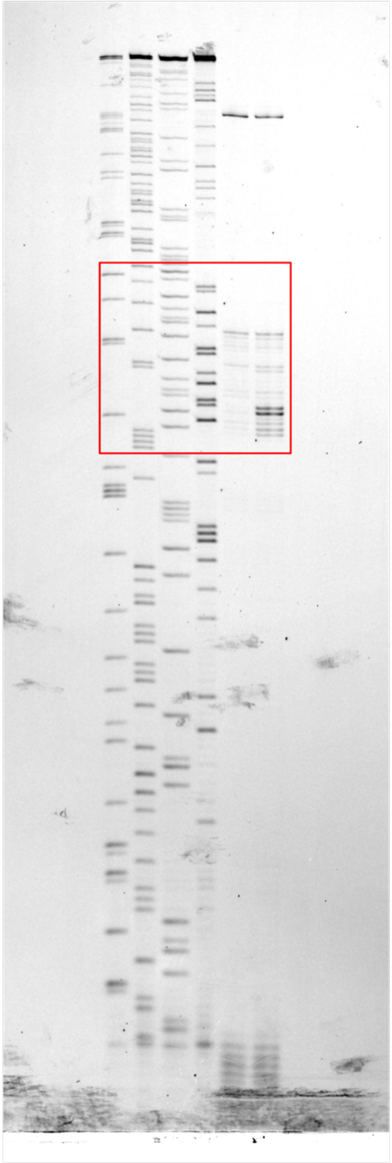

Supplement: Supplementary file 4 — Source Data [file 41467_2023_39553_MOESM4_ESM.zip › Source Data/381629_2_data_set_7719146_rvzjjv.pdf]
